# Hemodynamic monitoring using a single-use indwelling transesophageal echocardiography probe in an unstable patient after open-heart surgery

**DOI:** 10.1186/s12880-015-0070-3

**Published:** 2015-08-14

**Authors:** Emmanuelle Begot, Marc Clavel, Alessandro Piccardo, Rémi Bellier, Bruno François, Nicolas Pichon, Philippe Vignon

**Affiliations:** Medical-surgical intensive care unit, Dupuytren University Hospital, 2 Ave. Martin Luther King, 87042 Limoges, France; Inserm, CIC-1435, F-87000 Limoges, France; Department of Thoracic and Cardiovascular Surgery, Dupuytren University Hospital, Limoges, France; Université de Limoges, F-87000 Limoges, France

**Keywords:** Monitoring, Transesophageal echocardiography, Patent foramen ovale, Tamponade, Mediastinal hematoma

## Abstract

**Background:**

Hemodynamic monitoring is frequently needed in ventilated patients with unstable hemodynamics after open-heart surgery. Novel miniaturized single-use transesophageal echocardiographic probe has been scarcely used in this clinical setting.

**Case presentation:**

A patient who underwent a scheduled open-heart surgery developed a ventilator-associated pneumonia and was referred to the intensive care unit for post-operative acute respiratory distress syndrome. Hemodynamic monitoring was performed with a single-use indwelling transesophageal echocardiography probe during 50 h. Initially, a contrast study depicted a patent foramen ovale with a right-to-left shunt. Nitric oxide was administered and positive end-expiration pressure was reduced. Subsequently, the patient became hemodynamically unstable and the identification of a localized tamponade due to compressive left atrial hematoma prompted reoperation.

**Conclusions:**

The novel hemodynamic monitoring device described here appears valuable to help identifying severe post-operative complications and guide acute care.

## Background

Although multiplane transesophageal echocardiography (TEE) is increasingly used in intensive care unit (ICU) settings, it is not ideally suited for serial hemodynamic assessment of unstable ventilated patients. A commercially available miniaturized single-use 72-h indwelling TEE probe appears promising for the hemodynamic monitoring of ventilated ICU patients [1], as illustrated in the present case report.

## Case presentation

A 57-year-old patient with multiple cardiovascular risk factors was hospitalized for an uncomplicated anterolateral non-ST elevation acute coronary syndrome. He was treated with Aspirin, unfractionned Heparin and Prasugrel. The coronary angiography showed a three-vessel disease of the left anterior descending, lateral and right coronary arteries. Left ventricular ejection fraction (LVEF) was 56 % with a mild anterolateral hypokinesis. Triple coronary artery bypass grafting was scheduled. The procedure was prolonged by intraoperative bleeding which was difficult to control (extracorporeal circulation: 4 h16; aortic clamping: 3 h13). Postoperative TEE disclosed a moderately decreased LVEF of 45 % with an anterolateral hypokinesis, normal left ventricular (LV) filling pressures and right ventricular (RV) function. A vasopressor support (1.6 mg/h) was required due to unstable hemodynamics (lactate: 4.28 mmol/L). On Day 4, a ventilator-associated pneumonia was diagnosed (*Klebsiella pneumoniae* and *Hemophilus influenzae*). Despite adapted antibiotic therapy, the patient developed a severe acute respiratory distress syndrome (ARDS) on Day 7, with a PaO_2_/FiO_2_ ratio of 74 (FiO_2_: 1).

**Fig. 1:**
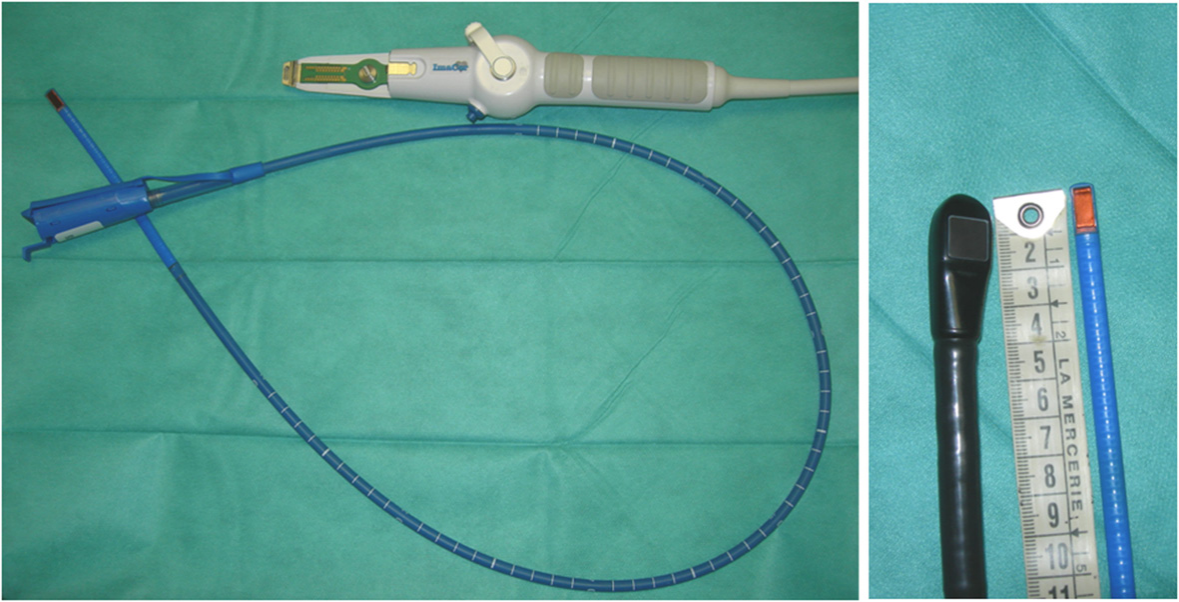
Single-use miniaturized transesophageal echocardiographic probe. The single-use indwelling monoplane transesophageal echocardiography probe can be left in place for up to 72 h and is easily connected to a dedicated system for serial hemodynamic assessments (*left panel*). Its small size compared to regular multiplane transesophageal echocardiographic probes facilitates hemodynamic monitoring (*right panel*)

**Fig. 2:**
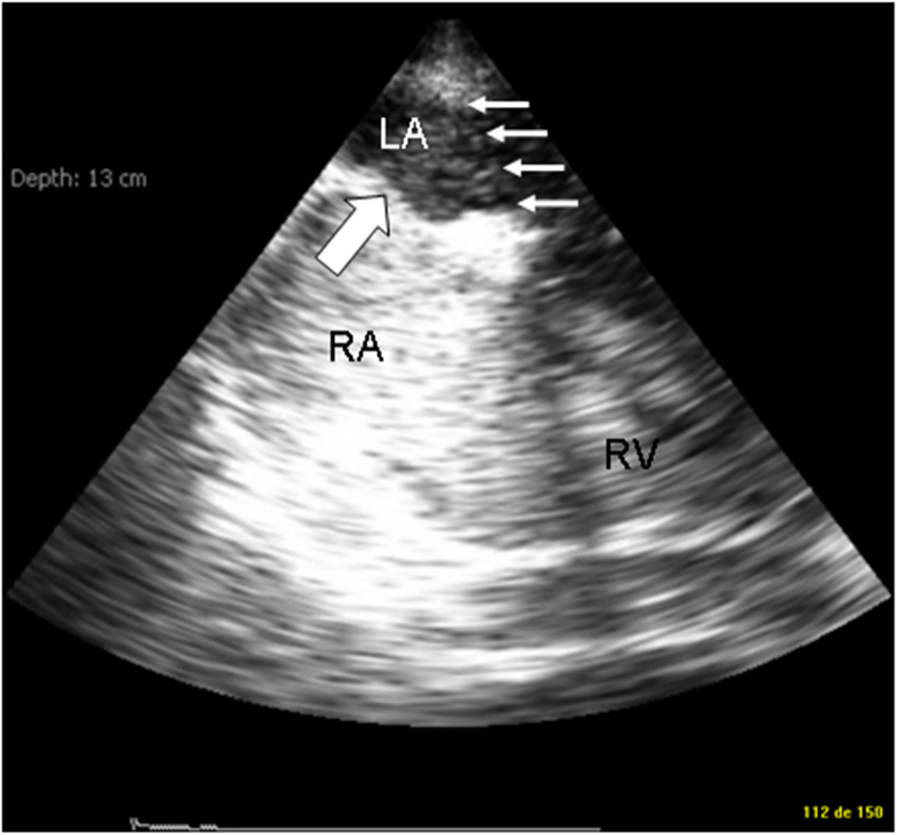
Patent foramen ovale revealed by a contrast study. The injection of agitated saline fully opacified the moderately dilated right atrium and underlined a septal aneurysm bulking towards the left atrium at end-expiration (*thick arrow*). A large shunting through a patent foramen ovale with full opacification of the left atrium was evidenced in this patient with severe acute respiratory distress syndrome (*thin arrows*). Abbreviations: LA, left atrium; RA, right atrium; RV, right ventricle

**Fig. 3:**
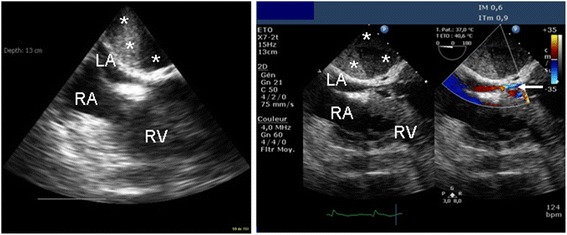
Extrapericardial tamponade. The miniaturized transesophageal echocardiographic probe disclosed a large mediastinal hematoma compressing the left atrium in this patient with severe shock (*left panel, asterisks*). The presence of a localized tamponade was confirmed during conventional transesophageal echocardiography which depicted a rounded heterogeneous mass consistent with a recent hematoma which compressed the left atrium and impaired atrial filling (*mid panel, asterisks*), as reflected by a narrow and turbulent inflow on color Doppler mapping (*right panel, arrow*). Abbreviations: LA, left atrium; RA, right atrium; RV, right ventricle

Hemodynamic monitoring was initiated on Day 6 due to persistent need of vasopressor support and worsening of gas exchange. It was performed using a single-use, monoplane TEE miniaturized probe (diameter 5.5 mm) connected to a dedicated echographic system which allows two-dimensional imaging and color Doppler mapping, but has no spectral Doppler capability (Imacor®, New-York, NY, USA). This device is approved by the Food and Drug Administration and has a European Community mark for 72 h of continuous use (Fig. 1). The patient was hemodynamically assessed every 6 to 8 h and whenever a clinically relevant event occurred. The transverse view of great vessels, long axis view and transgastric short-axis view of the heart were analyzed to determine the main mechanism that caused circulatory failure [1]. The first hemodynamic evaluations ruled out a preload-dependence, a relevant ventricular dysfunction and valvulopathy, and a pericardial effusion (Table 1). Specifically, there was no evidence for an early surgery-related complication such as mediastinal hematoma. Accordingly, the vasopressor support was maintained since patient’s hemodynamic status was stabilized with steady infusion rates. Despite a protective ventilation and a PEEP trial (FiO_2_: 1; respiratory rate: 22/min; tidal volume: 6 ml/kg ideal body weight; PEEP: 14 cmH_2_O), the PaO_2_/FiO_2_ ratio remained as low as 63. Accordingly, a contrast study was performed at H37 to screen for a potential patent foramen ovale (PFO). A PFO shunting was depicted with an atrial septum aneurysm (Fig. 2). PEEP was decreased to 8 cm H_2_O and inhaled nitric oxide was administered while the patient remained hemodynamically stable under vasopressor support (Table 1). This resulted in an increase of the PaO_2_/FiO_2_ ratio up to 130. At H46, the clinical course was complicated by an abrupt hemodynamic deterioration with hypotension, skin mottling and oliguria associated with a rapid supraventricular tachycardia (170 bpm), which was efficiently treated by anti-arrhythmic drugs. A new hemodynamic assessment using the miniaturized TEE probe disclosed a large mediastinal hematoma compressing the entire left atrium (LA) and a moderate, non compressive pericardial effusion. To ascertain the diagnosis of localized tamponade prior to surgical decompression, a regular multiplane TEE study was immediately performed and was confirmatory (Fig. 3).

**Table 1:** Hemodynamic monitoring using the single-use 72-h indwelling transesophageal echocardiography probe^a^

	H0	H5	H14	H22	H28	H37	H46	H50
Variations of SVC size	Moderate	Moderate	Moderate	None	None	None	None	None
LV fractional area change	Normal	Normal	Normal	Normal	Normal	Normal	Decreased	Normal
RV dilatation	Absence	Absence	Absence	Absence	Absence	Absence	Absence	Absence
Paradoxical septal motion	Absence	Absence	Absence	Absence	Absence	Absence	Absence	Absence
Severe left-sided valvular regurgitation	Absence	Absence	Absence	Absence	Absence	Absence	Absence	Absence
Other relevant abnormality	No	No	No	No	No	PFO shunting	Posterior mediastinal hematoma compressing the left atrium	No
Therapeutic impact	Vaso-pressor	Vaso-pressor	Vaso-pressor	Vaso-pressor	Vaso-pressor	Reduce PEEP Nitric oxide	Emergency surgical evacuation of left atrial hematoma	Vaso-pressor

*SVC* superior vena cava, *LV* left ventricle, *RV* right ventricle, *PEEP* positive end-expiratory pressure

^a^In the presence of a circulatory failure, the following therapeutic algorithm based on the analysis of three transverse views (great vessels, transesophageal long-axis four-chamber view of the heart, transgastric short-axis view of the heart) was used: large respiratory variations of superior vena cava size (inspiratory collapse) in the transverse view of the great vessel were indicative of preload-dependence (fluid loading); a right ventricular end-diastolic area exceeding the left ventricular end-diastolic area in the transesophageal long-axis four-chamber view of the heart was indicative of a marked dilatation of a failing right ventricle, potentially associated with an acute cor pulmonale; left ventricular fractional area change < 45 % in the transgastric short-axis view of the heart was indicative of systolic dysfunction in the absence of preload-dependence (administration of inotropes), while a paradoxical septal motion with a restrained left ventricle in this view was indicative of acute cor pulmonale (protective ventilation, reduced PEEP level, prone ventilation, nitric oxide administration, vasopressor support)

The patient was immediately reoperated. The surgeon confirmed the presence of a non compressive hemopericardium of ~300 mL and of a tamponade secondary to a large LA hematoma of ~500 mL of fresh blood. The patient sustained shock with multiple organ failure. Further TEE assessment confirmed the full evacuation of LA hematoma and ruled out a preload-dependence or a ventricular dysfunction. Despite the increase of vasopressor therapy, the patient died on Day 3 secondary to refractory shock.

## Discussion and conclusions

In our unstable ventilated patient with cardiorespiratory compromise, the novel single-use TEE probe was left in place for 50 h and allowed 8 hemodynamic assessments, two of which had a direct therapeutic impact, including urgent reoperation. In a recent pilot study, image quality was deemed adequate or optimal in more than 90 % of ventilated ICU patients and directly influenced therapy in 52 out of 94 patients (68 %) [1]. Long-term esophageal intubation by the single-use 72-h indwelling TEE probe was complicated by minor self-limited gastric bleeding (*n* = 2) and lip mechanical ulceration (*n* = 2), but none of these events had clinical consequences [1].

In our patient, TEE hemodynamic monitoring confirmed that initial therapy based on vasopressor support was adequate since no preload-dependence or ventricular dysfunction was observed [2]. It also ruled out an acute cor pulmonale which occurs in approximately 22 % of patients with moderate-to-severe ARDS despite protective ventilation [3–5]. Since ARDS patients with PFO shunting are poor responders to PEEP trials [4], we performed a contrast study which has been reported to be positive in 15.5 to 19.2 % of ventilated ARDS patients [3, 4]. Decreased PEEP level and nitric oxide administration improved hypoxemia, presumably in reducing RV afterload. Our patient was not ventilated in prone position because of unstable hemodynamics and recent sternotomy.

As the hemodynamic status was deteriorating, TEE monitoring disclosed a LA hematoma which developed within a few hours and would presumably have been missed otherwise, since typical tamponade physiology is uncommon after open-heart surgery [6]. The miniaturized TEE probe depicted an echodense, rounded mass which compressed the LA, with an inverted free wall curvature and turbulent intracavitary blood flow on Doppler color flow mapping [6]. We confirmed the diagnosis using conventional TEE because it remains the gold standard due to the poor diagnostic accuracy of transthoracic echocardiography [6, 7]. Despite emergency reoperation, the patient developed lethal refractory shock. This complication results in a mortality rate of 22 % for this type of localized tamponade despite prompt surgical evacuation [6].

In closing, hemodynamic monitoring using a single-use miniaturized TEE monoplane probe allows the identification of postoperative complications in patients with cardiopulmonary compromise after open-heart surgery. The diagnostic capability of this new device remains to be confirmed by further studies.

## Consent

Written informed consent was obtained from the next of kin of the patient for publication of this case report and any accompanying images. A copy of the written consent is available for review by the Editor of this journal.

## Abbreviations

ARDS: Acute respiratory distress syndrome
ICU: Intensive care unit
LA: Left atrium
LV: Left ventricle
LVEF: Left ventricular ejection fraction
PEEP: Positive end-expiratory pressure
PFO: Patent foramen ovale
RV: Right ventricle
TEE: Transesophageal echocardiography
